# Quantitative magnetic resonance imaging of microvascular changes in KHT sarcoma

**DOI:** 10.1186/1470-7330-14-S1-P13

**Published:** 2014-10-09

**Authors:** FT Kurz, HP Schlemmer, S Heiland, M Bendszus, CH Ziener

**Affiliations:** 1 University Hospital Heidelberg, German Cancer Research Center, Germany

## 

Micromorphological changes in skeletal muscle vascular remodeling due to denervation, aging or tumour growth have been investigated recently. Naturally, quantitative imaging of the capillary bed and the determination of microstructural parameters in healthy and pathological tissue might prove beneficial for potential therapeutic interventions. Susceptibility differences of capillaries and surrounding tissue are due to paramagnetic deoxyhaemoglobin and can be used to provide a means of quantifying microstructural parameters through MRI transverse relaxation time measurements. Within a local weak field approximation and based on simple geometrical assumptions, a model is introduced that analytically derives a dependence of blood transverse relaxation rate for Carr-Purcell-Meiboom-Gill sequences on inter-pulse delay time, strength of the magnetic field, local susceptibility difference and spin diffusion constant, capillary radius and local blood volume fraction. The model is tested for healthy skeletal muscle tissue in mouse leg muscle and KHT sarcoma based on experimental *ex-vivo* experiments. Due to blood loss and increased vessel wall permeability after excision, a capillary shrinkage to about 1/10 of the typical in-vivo capillary diameter of 1.5 µm is assumed, and, in a magnetic field of 0.6 T, the corresponding local diffusion constant in healthy tissue is found to be 2.2 µm2/ms, and for KHT sarcoma 2.6 µm2/ms. Therefore, the quantitative model might be used to differentiate between healthy and pathological tissue diffusion properties or, by assuming local diffusive properties, to determine differences in local microvasculature.

